# HOME CARE WORKER PERSPECTIVES ON PERSON-CENTERED CARE FOR PEOPLE WITH DEMENTIA

**DOI:** 10.1093/geroni/igae098.1234

**Published:** 2024-12-31

**Authors:** Jennifer Reckrey, Emily Xu, Deborah Watman, Sasha Perez, Emily Franzosa

**Affiliations:** Icahn School of Medicine at Mount Sinai, Icahn School of Medicine at Mount Sinai, New York, United States; Icahn School of Medicine at Mount Sinai, New York, New York, United States; Icahn School of Medicine at Mount Sinai, New York, New York, United States; Icahn School of Medicine at Mount Sinai, New York, New York, United States; James J Peters VA Medical Center, Bronx, New York, United States

## Abstract

Person-centered care for people living with dementia has been associated with improved functional ability and quality of life, yet little is known about person-centered care in the home setting. This study explored home care worker perspectives on providing person-centered care for people living with dementia. Using secondary qualitative analysis of 22 semi-structured interviews with home care workers, we identified themes related to the Dementia Initiative’s person-centered dementia care framework. We found that home care workers frequently acknowledged their client’s personhood and developed meaningful relationships with their clients, implementing individualized strategies to meet client needs. However, barriers to person-centered care included limitations of home care worker scope of practice and challenging dynamics with other home care workers and family caregivers. In addition, home care agencies’ formal care plans sometimes served as a barrier to person-centered care, but home care workers advocated within and around their task-based duties to ensure client needs were met. This analysis highlights the importance of integrating home care workers in person-centered healthcare teams and sustaining the meaningful relationships between home care workers and their clients, family caregivers, and other home care workers. Standardized training of home care workers in the principles and goals of person-centered dementia care could enhance existing person-centered care practices and promote alternatives to disease-centered practices. Recognizing home care workers’ unique role in providing person-centered care is essential to identify and meet the needs of people living with dementia in the community and their family caregivers.

